# BAP和β-CTX与肺癌骨转移进展程度的相关性

**DOI:** 10.3779/j.issn.1009-3419.2013.03.05

**Published:** 2013-03-20

**Authors:** 琼 唐, 辉 赵, 锐 贾, 林林 刘

**Affiliations:** 300121 天津，天津市人民医院呼吸内科 Department of Respiratory Medicine, Tianjin Union Medicine Center, Tianjin 300121, China

**Keywords:** 生化指标, β-CTX, BAP, 肺肿瘤, 骨转移, Biological markers, β Isomer of the C-terminal telopeptide of type Ⅰ collagen, Bone-specific alkaline phosphates, Lung neoplasms, Bone metastasis

## Abstract

**背景与目的:**

肺癌易发生骨转移，Ⅰ型胶原羧基端肽β特殊序列（β isomer of C-terminal telopeptide of type Ⅰ collagen, β-CTX）和骨源性碱性磷酸酶（bone-specific alkaline phosphates, BAP）在骨质代谢中是重要的生化标记物，本研究旨在探讨骨代谢生化指标和肺癌骨转移进展程度的相关性，有助于早期判断是否有肺癌骨转移的发生。

**方法:**

天津市人民医院2009年7月-2012年7月共收治92例肺癌并发生骨转移患者，全部病例其原发灶均经细胞学检查证实，骨转移灶的数目及类型经ECT结合X线片、CT证实，采用ELISA方法检测血β-CTX、BAP的浓度水平。

**结果:**

收集的92例肺癌伴骨转移的患者中，转移灶 < 3处为58例，≥3处为34例；溶骨型68例，成骨型9例，混合型15例。血β-CTX、BAP水平在不同的骨转移数目组间差异有统计学意义（*P* < 0.05），β-CTX在肺癌溶骨型骨转移患者中具有较高的敏感性。

**结论:**

骨代谢生化指标β-CTX、BAP对肺癌骨转移具有辅助诊断意义，是判断转移进展程度的良好指标。

## Abstract (en)

**Background and objective:**

Bone metastasis is common in lung cancer patients. The β isomer of the C-terminal telopeptide of type Ⅰ collagen (β-CTX) and bone-specific alkaline phosphates (BAP) are regarded as important bone turnover markers in bone resorption and formation. Thus, the aims of this study are to determine the correlation of these bone turnover markers with the extent of bone metastasis of lung cancer.

**Methods:**

A total of 92 patients with bone metastasis of lung cancer from Tianjin Union Medicine Center were enrolled in this study between July 2009 and July 2012. The bone metastasis lesion was diagnosed by ECT combined with X-ray and CT imaging. The bone turnover marker levels of β-CTX and BAP were measured by ELISA.

**Results:**

Among the 92 patients, 58 cases had < 3 bone-metastasis number and 34 had ≥3; 68 cases had osteolytic bone metastasis, 9 had osteoblastic bone metastasis, and 15 had mixed bone metastases. The serum levels of β-CTX and BAP were significantly higher in patients with >3 bone-metastasis number than that in patients with less than 3 (*P* < 0.05). β-CTX was more sensitive than BAP in diagnosing lytic bone metastasis.

**Conclusion:**

β-CTX and BAP can be used as supplementary tools for diagnosing and screening bone metastasis of lung cancer.

肺癌是常见的恶性肿瘤之一，肺癌易发骨转移，临床转移发生率约为30%-55%^[1]^。肺癌骨转移不仅缩短了患者的生存时间，而且疼痛、活动严重受限等并发症导致患者生存质量明显降低。肺癌发生骨转移后会引起骨质的代谢紊乱，发生骨质破坏和再形成等骨质代谢变化，骨代谢的生化指标可有助于早期判断肺癌的骨转移，并监测转移的进展程度，对判断骨转移的病情发展有一定的临床意义^[2]^。

## 1 材料与方法

### 1.1 研究对象及方法

回顾性分析2009年7月-2012年7月在天津市人民医院呼吸内科和脊柱外科接受治疗的肺癌骨转移患者。根据细胞学分析确诊肺癌及判断类型，根据放射性核素显像（emission computed tomography, ECT）及X片、CT确诊骨转移及转移类型和数目^[3]^，采用ELISA方法检测血，Ⅰ型胶原羧基端肽β特殊序列（β isomer of C-terminal telopeptide of type Ⅰ collagen, β-CTX）、血清骨源性碱性磷酸酶（bone-specific alkaline phosphates, BAP）的浓度水平。研究对象入选标准：①细胞学确诊肺癌患者；②经过ECT、X线平片、CT检查确诊发生骨转移；③采用ELISA方法测定患者的骨代谢生化指标；④随访时间至少2个月。排除标准：①以上资料不全的病例均被排除；②风湿性或骨性关节炎、明确的骨质疏松症、甲状旁腺功能亢进者排除。

### 1.2 统计学处理

采用SPSS 10.0软件进行统计分析，各骨代谢生化指标在不同组间的比较采用秩和检验，*P* < 0.05为差异有统计学意义。

## 2 结果

### 2.1 病例临床特征

共92例肺癌骨转移患者符合入组标准，其中男55例，女37例；平均年龄56岁（39岁-73岁）；腺癌48例（52.17%），鳞癌25例（27.17%），小细胞肺癌12例（13.04%），混合型7例（7.62%）；骨转移数目 < 3为58例（63.04%），转移数目≥3为34例（36.96%）；溶骨型68例（73.91%），成骨性9例（9.79%），混合型15例（16.30%）。

### 2.2 肺癌骨转移数目及骨转移类型与各指标相关度的比较

见表 1。β-CTX和BAP浓度水平随骨转移程度增加而增加，在骨转移部位≥3处的浓度较骨转移部位 < 3处明显升高（*P* < 0.05）。β-CTX和BAP的浓度在溶骨型转移组、成骨型转移组、混合型转移组三组间比较无明显统计学差异（*P*>0.05）。

**1:** Ⅰ型胶原羧基端肽*β*特殊序列和骨源性碱性磷酸酶与肺癌骨转移部位的数目及骨转移类型的相关分析 The correlation between *β* isomer of C-terminal telopeptide of type Ⅰ collagen (*β*-CTX) and bone-specific alkaline phosphates (BAP) with the number and character of metastatic loci in bone

Characteristic	*n*	BAP (μg/L)		*P*	*β*-CTX (ng/mL)		*P*
		Median	Range		Median	Range	
Number of bone metastases in bone				0.025			0.01
< 3	58	31.20	25.15-37.25		1.515	0.804-1.873	
≥3	34	57.37	35.28-79.46		2.520	1.025-3.015	
Character of bone metastases				>0.05			>0.05
Osteolytic	68	37.19	25.15-46.25		1.426	0.932-2.015	
Osteoblastic	9	41.67	33.43-79.46		1.094	0.915-1.569	
Mixed	15	39.27	27.32-66.28		1.256	0.973-1.682	

### 2.3 β-CTX在诊断肺癌溶骨性骨转移中的敏感度及特异度

见表 2。β-CTX在溶骨性肺癌骨转移的敏感度为76.5%（52/68），特异度为62.5%（15/24）。

**2:** *β*-CTX在诊断肺癌溶骨性骨转移中的敏感度、特异度 The sensitivity and specificity of *β*-CTX in the diagnosis of osteolytic bone metastasis in lung cancer

Osteolytic bone metastasis	*β*-CTX		Total
	Positive	Negative	
Positive	52	16	68
Negative	9	15	24
Total	67	25	92

## 3 讨论

### 3.1 肺癌骨转移的诊断

肺癌骨转移以ECT及X线平片方法确诊，一般认为ECT可较X线平片提前3个月-6个月发现异常信号，但对单发性病灶有一定假阳性。X线平片及CT检查一般无假阳性，但X线检出病变取决于病变脱钙和钙质沉积引起骨密度变化的程度，只有局部钙的丢失大于30%-50%及病灶>2 cm时，X线才能发现病灶，其敏感性及特异性较ECT和MRI低^[4, 5]^。而且通过影像学检查不仅花费昂贵，敏感性低，还有辐射的危害。目前研究表明，骨代谢生化指标检测骨转移的敏感性、特异性较高，对协助诊断肺癌骨转移程度具有辅助诊断价值。本组研究中，92例肺癌骨转移患者中75例表现为β-CTX水平高于正常，其中部分骨扫描和影像学均为阴性。但随后的2个月随访中影像学检查证实了骨质缺失的诊断。β-CTX在溶骨性肺癌骨转移早期的敏感率达到70.6%，表明它是骨转移进展程度的一个有敏感的监测指标。

### 3.2 骨生化代谢指标BAP、β-CTX与肺癌骨转移相关度

骨代谢生化指标包括骨形成和骨吸收生化指标，本组收集的病例中溶骨型肺癌骨转移占73.91%（68/92），这与既往报道肺癌骨转移主要以多发型、溶骨型骨破坏为特征相一致^[3]^。过去的几年在恶性肿瘤骨转移的临床诊断和随访治疗中，骨质代谢的新的生化标记物已经得到了广泛的研究。孔清泉等^[6]^在其研究报道中认为血清BAP和CTX可监测早期肺癌骨转移的发生。Ⅰ型胶原吡啶交联终肽（cross-linked C-terminal telopeptide of type Ⅰ collagen, ICTP）和Ⅰ型胶原羟基末端肽β特殊序列（β-CTX）是由Ⅰ型胶原肽羧基末端降解而来，是其中最常见的骨吸收标志物。ICTP的抗原表位紧邻CTX，但属胶原酶MMP（matrix metalloproteinase）激活引起的骨质释放已被证明是肺癌骨转移的标记物，且与骨质病变部位的数量呈正相关，是肺癌骨转移进展程度的良好指标^[7]^。通过组织蛋白酶K裂解Ⅰ型胶原产生的CTX及其在骨质代谢中的释放率是反映破骨细胞骨吸收活动的一个有用的指标。在新合成的Ⅰ型胶原蛋白羧基末端肽是α-型（α-CTX），而在成熟Ⅰ型胶原蛋白的羧基末端肽主要是β-型（β-CTX）^[8]^。本次研究发现，骨转移肺癌患者的血清中β-CTX浓度与骨转移的部位及数目正相关，结果具有统计学意义。92个肺癌骨转移患者中，62个表现为β-CTX水平高于正常，但是骨扫描和影像学均为阴性。随后的2个月随访中影像学检查证实了骨质缺失的诊断。这一结果表明，在肺癌骨转移早期阶段，β-CTX的水平可能会升高，此时骨扫描和骨显像无法探测。骨转移数目与血β-CTX浓度相关性具有统计学意义，这表明血液β-CTX是肺癌骨转移程度的敏感指标。

血清总碱性磷酸酶分别由肝脏及成骨细胞合成（几乎各为50%），均是同工酶，血清总碱性磷酸酶的表达程度可以反映骨的形成进程，BAP主要由成骨细胞分泌，能够反映成骨细胞的功能变化。在成骨细胞发育过程中，是成骨细胞早期分化的标志，是特异的骨形成指标。实验结果显示：骨转移个数≥3处组比转移个数 < 3处组的BAP浓度水平明显升高，有统计学差异。与溶骨型肺癌骨转移组相比较，成骨型骨转移组BAP中位数水平增加，但差异无明显统计学意义。另外2篇文献作者^[9, 10]^也认为肺癌骨转移患者与非转移患者相比BAP水平变化无明显相关性。肺癌骨转移引起的骨质代谢过程中成骨细胞活性和破骨细胞活性均增强，这和两者之间相互作用有关。β-CTX和BAP在溶骨型骨转移和成骨型骨转移中均增加说明骨转移发生过程中成骨细胞和破骨细胞作用是相互的，此观点与以前的研究^[11, 12]^报告一致。

本组报告中，β-CTX在溶骨性肺癌骨转移早期的敏感率达到76.5%，而特异度为62.5%，表明它是骨转移进展程度的一个较敏感的监测指标。在筛查或诊断在溶骨型骨转移肺癌中，骨吸收标志物β-CTX比骨形成的标志物BAP具有更重要的临床意义和适用价值。本实验仍有以下局限，样本量有限，属于回顾性分析研究，希望今后会有大样本前瞻性对照研究来进一步检验与肺癌骨转移进展相关性。虽然β-CTX和BAP尚不能代替骨扫描和骨影像学检查，骨质转换生化标记已为临床医生提供一种新的辅助诊断工具，也是筛查高危患者的一个重要指标。
